# Preparation of active 3D film patches *via* aligned fiber electrohydrodynamic (EHD) printing

**DOI:** 10.1038/srep43924

**Published:** 2017-03-08

**Authors:** Jun-Chuan Wang, Hongxia Zheng, Ming-Wei Chang, Zeeshan Ahmad, Jing-Song Li

**Affiliations:** 1 Key Laboratory for Biomedical Engineering of Education Ministry of China, Hangzhou, 310027, P. R. China; 2 Zhejiang Provincial Key Laboratory of Cardio-Cerebral Vascular Detection Technology and Medicinal Effectiveness Appraisal, Hangzhou, 310027, P. R. China; 3 Hangzhou Dental Hospital, Hangzhou, 310006, P. R. China; 4 Leicester School of Pharmacy, De Montfort, University, The Gateway, Leicester, LE1 9BH, UK

## Abstract

The design, preparation and application of three-dimensional (3D) printed structures have gained appreciable interest in recent times, particularly for drug dosage development. In this study, the electrohydrodynamic (EHD) printing technique was developed to fabricate aligned-fiber antibiotic (tetracycline hydrochloride, TE-HCL) patches using polycaprolactone (PCL), polyvinyl pyrrolidone (PVP) and their composite system (PVP-PCL). Drug loaded 3D patches possessed perfectly aligned fibers giving rise to fibrous strut orientation, variable inter-strut pore size and controlled film width (*via* layering). The effect of operating parameters on fiber deposition and alignment were explored, and the impact of the film structure, composition and drug loading was evaluated. FTIR demonstrated successful TE-HCL encapsulation in aligned fibers. Patches prepared using PVP and TE-HCL displayed enhanced hydrophobicity. Tensile tests exhibited changes to mechanical properties arising from additive effects. Release of antibiotic from PCL-PVP dosage forms was shown over 5 days and was slower compared to pure PCL or PVP. The printed patch void size also influenced antibiotic release behavior. The EHDA printing technique provides an exciting opportunity to tailor dosage forms in a single-step with minimal excipients and operations. These developments are crucial to meet demands where dosage forms cannot be manufactured rapidly or when a personalized approach is required.

Polymeric dosage forms are amongst the most widely researched drug delivery systems. Based on polymer structure and fabrication; drug release kinetics, distribution and absorption are tailored to improve efficacy and safety^1^. In recent times, novel approaches in polymeric drug dosage form fabrication and engineering have led to the development of new pharmaceutical technologies^2^,^3^. The utilization and development of 3D micrometer and nanometer scale systems for emerging therapies (e.g. tissue engineering scaffolds^4^) and conventional drug delivery^5^ elucidates the convergence of interdisciplinary concepts and strategies to address several healthcare challenges. Within this advance, the maturing electrospinning (ES) technique has been explored extensively for fibrous (or filamentous) drug delivery^6^. However, in the vast majority of cases, ES is limited to ‘random’ 2D structure fabrication, and where fiber alignment has been demonstrated (e.g. using grounded substrates^7^) resulting structures clearly lack directional precision. Furthermore, complex alignment (e.g. multi-axial patterns) using such methods is scarce.

Randomly orientated 2D drug-loaded fibers (engineered *via* ES) generally display a broad diameter range. This leads to greater deviation in drug release kinetics between sample batches. There is also inadequate control on modulating interstitial pore size within fibrous films when prepared using the ES process^7^. It is well established that porous surfaces impede drug release from matrix systems (i.e. through diffusion) when in contact with simulated biological fluids^8^. In this regard the ES technique is restricted (e.g. drug loading and film thickness), as interstitial pore volume reduces with increasing fiber deposition time. Furthermore, electrospun drug-polymer films need to be cut or shaped into the desired dosage form geometry and since the process inherently involves repulsion (electrostatic); a clear difference in fiber diameter distribution and film thickness across the substrate plane is often observed^9^. One parameter frequently explored in ES is the deposition distance and this is also known to influence structure morphology, filament diameter size and phase separation between formulated excipients during the fiber forming process^10^,^11^. Variations in these alter drug release behaviour from a matrix type system which in extremely undesirable for potential drug dosage forms. Hence, while the ES technique is facile, cost-effective and permits micron-scaled structure engineering; random deposition limits its fabrication applications where precision engineering is critical (for both matrix and porosity). Several strategies to align fibers have therefore been developed, ranging from parallel electrodes^12^ to printing^13^. The latter is ideal due to control precision, quantity deposited and complex structure engineering^14^. The development of drug dosage forms using controlled deposition *via* 3D printing is an emerging field; although to date limited studies have been explored using a derivative of the ES technique - electrohydrodynamic (EHD) printing^13^. Here, the benefits of ES (e.g. ambient temperature process, facile manufacturing and low shear rates) are enabled; however additional distinct advantages of EHD printing provide greater control on fiber forming and deposition. For example; the geometry of a dosage form can be printed in a single step to suit the anatomical location or age of patient, the quantity of deposited drug-matrix fiber is known, layering of structures permits drug loading and film thickness modulation, the strut size and inter-strut pore (void) can be controlled and there is less wastage since there is no need to dispose of peripheral mat components after film shaping (as is the case with the ES technique). Furthermore, general benefits of 3D printing are applicable to the EHD method and minimal excipients can be used, drug loading can be increased and dosage forms can be tailored and personalised^15^,^16^,^17^. In addition, aligned fibers have potential applications in cell guidance (*via* release of cell-signalling moieties), super-capacitors and active biological scaffolds (possessing bioactive properties)^8^,^9^,^10^ where micron scaled arrangements are critical for desirable biological functions and interactions.

Complex 3D assembly and pattering of structures has been demonstrated using a variety of methods (e.g. photolithography^18^, gas foaming^19^, phase separation^20^, fused deposition modeling^21^ and precision extrusion deposition^22^). However, most of these are limited due to low structural resolution, number of processing steps and drug leaching or active instability during forming (for drug delivery development). In contrast, the EHD printing process offers relatively high resolution, ambient temperature operation, one-step fabrication and potential complex 3D structure preparation^23^. Compared to its sister ES process, EHD printing deposits single fibers to fabricate pre-determined 3D structures on a layer-by-layer basis, and is made possible by shortening the working (deposition) distance from >10 cm to <10 mm. This permits precision patterning and morphology control of single fibers, and through overprinting; ‘ordered’ architectures are generated^24^. While the benefits of ES^25^ have led to extensive explorations of the technique in drug delivery; the process remains random and resulting mean fiber diameter’s exhibit a broad distribution. Nonetheless, numerous studies into topical and transdermal drug delivery have been conducted specifically for potential wound healing materials or transdermal patches prepared using ES^26^,^27^,^28^. Ideal wound dressing materials or patches are moderately hydrophilic, porous, biodegradable, mechanically strong and in some advanced systems medicated (to release embedded drug in controlled fashion *via* diffusion). Fibrous drug loaded patches have also been developed for buccal and ocular delivery^29^,^30^. Therefore, the potential to develop 3D printed fibrous patch systems (for various anatomical, indications and age groups) with greater control on drug loading, release and patch geometry is immense.

Polycaprolactone (PCL) is an FDA approved polymer and is known for its biodegradability, biocompatibility, thermal and chemical stability, appreciable mechanical properties and permeability^31^,^32^,^33^. PCL-drug composite nanofiber mats have been investigated as controlled release wound dressing materials^34^. Polyvinyl pyrrolidone (PVP) has been used as a rapidly dissolving matrix carrier for actives in several drug delivery system formulations^35^. PVP can be blended homogeneously with PCL, and is known for its good complexion, adhesion properties, physiological compatibility and low chemical toxicity^36^.

Tetracycline hydrochloride (TE-HCL) has been used as a therapeutic reagent for anti-inflammatory and antibacterial indications. Prolonged TE-HCL release from nanofibers and cellular regeneration benefits have also been shown in previous studies^25^. Its potential use to treat infections during wound healing is also established^37^. Although several TE-HCL medicated patches have been developed^38^,^39^. the potential to print be-spoke 3D aligned drug loaded fibrous films, with various strut-pore geometries has not been explored.

In this study, a range of antibiotic 3D polymeric patches with precisely aligned fibers as multi-layers are prepared using EHD printing. The aim of the study was to identify and determine key parameters influencing fiber alignment using EHD processing conditions (e.g. solution concentration, drug loading, collector speed and formulation flow rate) and to explore the impact of patch material composition (excipients) and print-void geometry on drug release. It is envisaged the 3D EHD printing method is flexible and able to meet dosage form requirements needed at various anatomical locations for personalized drug delivery and tailored active release.

## Results and Discussion

### Optimization of PCL/PVP solution concentration for 3D structure printing

**Figure 1:**
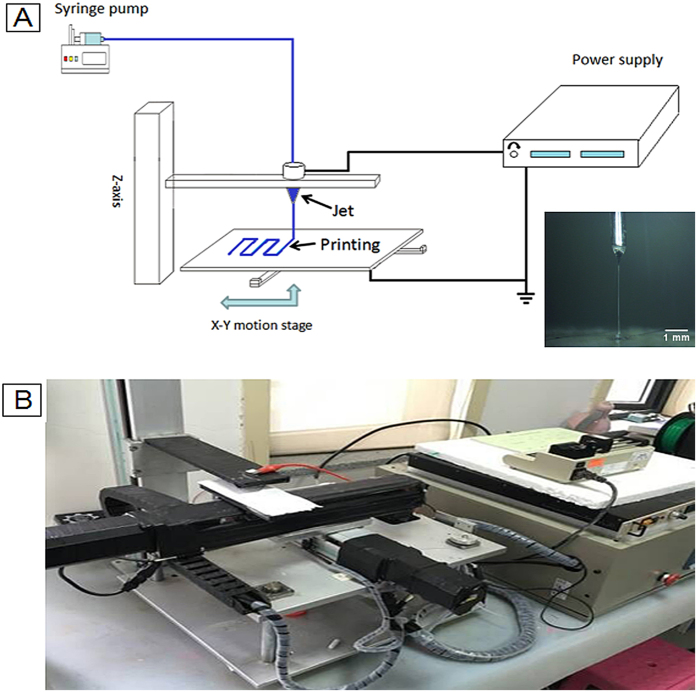
(**A**) Schematic of electrohydrodynamic (EHD) jet printing system and (**B**) digital image of the actual custom built EHD printing system.

PCL/PVP (w_PCL_/w_PVP_ = 2/1) solution concentration is critical for 3D micro-structure engineering *via* EHD printing. In conventional ES process, the polymer concentration in process formulation is generally low (typically, 1–10 w/v%). However, for the EHD printing process, fiber generation at such polymer concentrations is problematic. The ES process includes several stages during fiber formation (e.g. infusion, jetting, whipping & bending, collection and solidification). During EHD printing, many of these stages are bypassed or reduced (time) due to the relatively shorter working distance. This however provides an opportunity to control single fiber deposition in direct, continuous and controllable manner for micro-engineering *via* precise alignment of fibrous structures. Here, it is essential for fibers to solidify quickly over a short working distance (from nozzle tip to the collector substrate) which is required for pattern over-printing leading to solidified and well defined 3D structures. Therefore, the concentration of PCL/PVP solutions was kept in the range 20–30 w/v% for the present study and the effect of polymer concentration on 3D structure fabrication was also investigated. Figure 1 shows a schematic diagram of the EHD printing setup and an actual Taylor cone-jet obtained during EHD printing (as an inset).

**Figure 2:**
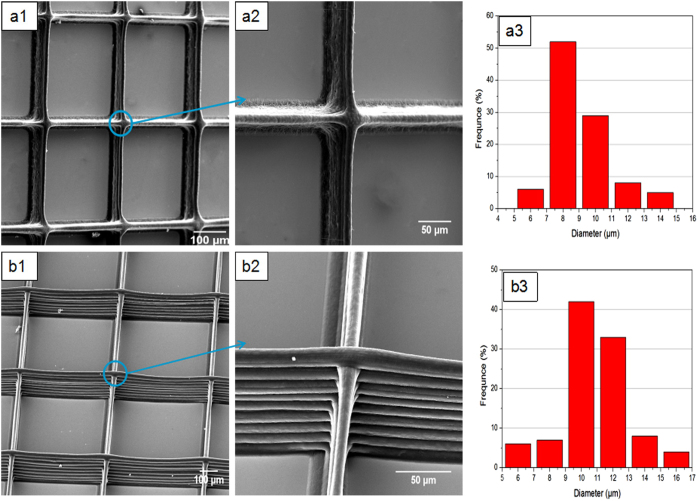
EHD printed films using different concentrations of PCL/PVP solution (**a**) 20 and (**b**) 25 w/w%.

Initially, the PCL/PVP solution concentration was maintained at 20 w/v%. The working distance, applied voltage, collector speed and flow rate were selected as 2 mm, 2 kV, 80 mm/s and 0.4 mL/h, respectively. Repeated over-printing, to prepare multi-layered 3D structures, was achieved as shown in Fig. 2a1. All fibers were deposited on the same location although aligned fibers merged together as shown in Fig. 2a2. Therefore, this solution concentration for EHD printing was deemed to be relatively low. In this instance, fiber merging is attributed to insufficient evaporation of the solvent, causing overprinted fibers to fuse at the central regions between perpendicular patterns. Figure 2a3 shows the mean printed fiber diameter to be 9.2 ± 1.2 μm when using 20 w/v % PCL/PVP solution.

In contrast, Fig. 2b1 shows uniform and smooth fibers which retain their morphology even during multi-layer deposition. For these, the PCL/PVP solution concentration was increased to 25 w/v%. Lack of fiber merging indicates improved solidification during the printing process. A 5 w/v% increase in polymer concentration (a 5% reduction in solvent) is sufficient for polymeric fibers to solidify rapidly during the EHD printing process over the short working distance (nozzle to collector), allowing deposited filamentous structures to maintain their integrity and morphology when in contact with adjacent fibers. The mean fiber diameter, however, increased to 11.2 ± 1.5 μm, which is attributed to an increase in solution viscosity which subsequently leads to a reduction in electrical conductivity^24^. Increasing the polymer concentration further to 30 w/w%, which also leads to an increase in viscosity and surface tension, provides parametric control on printed fiber width. However, at this concentration Taylor cone stability becomes compromised impacting fiber uniformity and continuity and this limits the process. Therefore for EHD 3D multi-layered fiber printing a PCL/PVP solution concentration of 25 w/w% was selected for further studies.

### Effect of conventional EHD parameters

**Figure 3:**
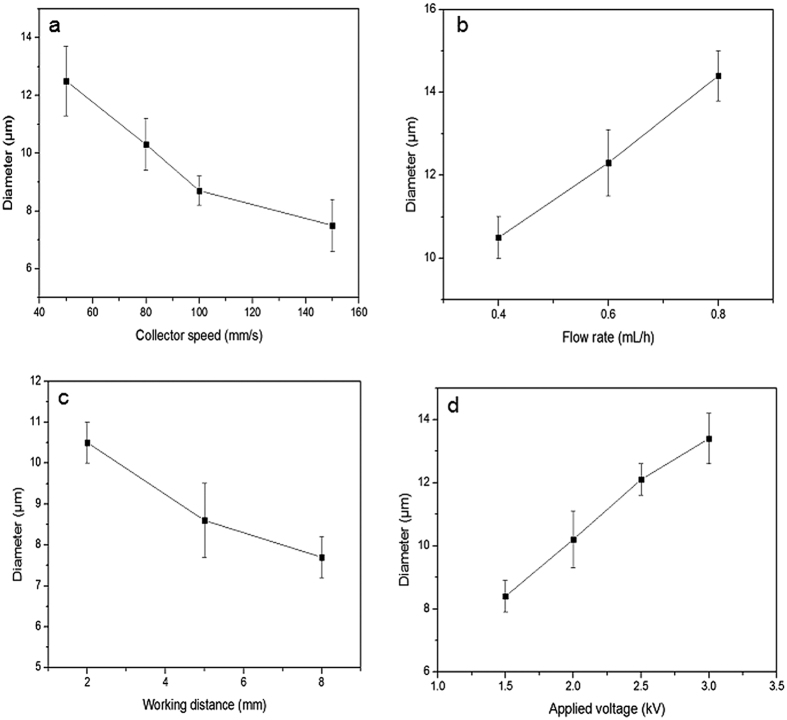
The effect of various operating parameters; (**a**) collector speed (**b**) flow rate (**c**) working distance and (**d**) applied voltage.

In the continuous EHD 3D printing process, fiber diameter is influenced by process material formulation (e.g. active(s) and excipients) and conventional EHD process parameters such as collector speed, applied voltage and working distance^23^. Therefore, the second phase of study involved determining ideal parameters for stable jetting and acceptable fiber generation (uniformity, continuity and mechanical integrity). EHD printed fibers were deposited using different collector speeds of 50, 80, 100 and 150 mm/s. The flow rate, working distance, applied voltage and PCL/PVP solution concentration were fixed at 0.4 mL/h, 2 mm, 2.0 kV and 25 w/w%, respectively. The mean diameter of resultant fibers decreased significantly from 12.5 ± 1.2 to 7.5 ± 0.9 μm (Fig. 3a) when the collector speed was increased from 50 to 150 mm/s. This is attributed to the change in drawing force, jet-substrate contact time and ejected jet spreading during EHD printing. There is potential for the jet to be stretched further on contact with the moving collector, especially when the collector speed is greater than the jet-deposition speed. Furthermore, the moving substrate has potential to reduce charge accumulation which in theory should improve EHD process stability.

Figure 3b shows the mean diameter of printed fibers based on flow rate variation (0.4–0.8 mL/h). Here, collector speed, working distance, applied voltage and PCL/PVP solution concentration were fixed at 80 mm/s, 2 mm, 2.0 kV and 25 w/v%, respectively. The mean fiber diameter increased with increasing flow rate from 10.5 ± 0.5 μm at 0.4 ml/h to 14.4 ± 0.6 μm at 0.8 ml/h. This is due to greater solution perfusion and, therefore, a greater volume of polymer within an elongated-jet. Excessive flow rate (>0.8 ml/h) becomes a limiting factor; interfering with uniform fiber production.

The deposition distance is a well-known parameter in conventional ES process^40^,^41^. When the collector speed, flow rate, applied voltage and PCL/PVP solution concentration were fixed at 80 mm/s, 0.4 mL/h, 2.0 kV and 25 w/v %, respectively, an increased working distance impacted fiber resolution. As shown in Fig. 3c, the fiber diameter decreased from 10.5 ± 0.5 to 7.7 ± 0.5 μm with an increase in working distance from 2 to 8 mm. This is due to enhanced polymer jetting and stretching, which also assists in the evaporation of base solvent, yielding printed fibers with reduced diameters. However, unstable jetting behavior becomes more pronounced with increasing working distance. which arises due to variance between jet deposition and collector speeds^42^.

The applied voltage is a crucial parameter in achieving stable jetting and fiber formation. As shown in Fig. 3d, the mean fiber diameter increased from 8.4 ± 0.5 to 13.4 ± 0.8 μm following incremental changes to the applied voltage (1.5 to 3 kV). Here, the collector speed, flow rate, working distance and PCL/PVP solution concentration was maintained at 80 mm/s, 0.4 mL/h, 2 mm, and 25 w/v%. Interestingly, during the ES process an increase in applied voltage results in fibers with reduced diameters. However, considering the reduced deposition (working) distance in EHD printing; at a higher working voltage the change in cone-geometry influences the exact location of cone and jet, leading to the collection of fibers closer to the cone, which is appreciably larger than the jet width.

### Multi-patterned drug composite fibers with various 3D aligned structures

The controlled patterning and layering of fibers has potential to engineer and tailor printed patches. In this instance, patches comprising drug-polymer filamentous matrices must be uniformly aligned and inter-fiber layered pores (or voids) must be assessed as these impact porosity and potentially drug release behavior.

**Figure 4:**
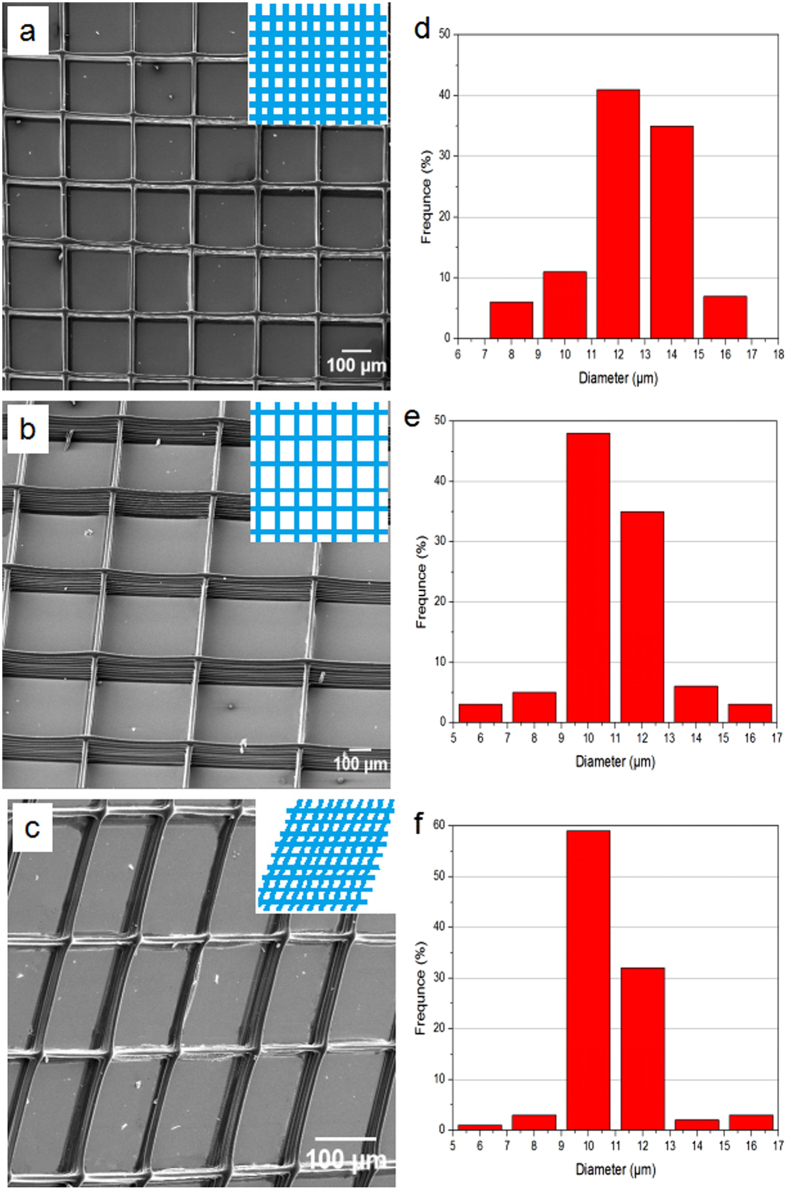
Various EHD printed 3D film patches showing (**a**) rectangular lattice patterns with grid pore size 200 × 200 μm^2^ (**b**) rectangular lattice patterns with grid pore size 500 × 500 μm^2^ and a (**c**) 3D rhomboid structure. Strut fiber diameter distribution of the (**d**) rectangular lattice patterns with grid pore size 200 × 200 μm^2^ (**e**) rectangular lattice patterns with grid pore size 500 × 500 μm^2^ and the (**f**) 3D rhomboid structure

Figure 4 shows electron micrographs of various 3D printed structures of PCL/PVP composite fibers generated using the EHD printing technique. Figure 4a and b show two well-aligned rectangular 3D structures possessing grid void sizes of 200 × 200 and 500 × 500 μm^2^. For the latter, the solution concentration, working distance, applied voltage, flow rate and collector speed were maintained at 25 w/v%, 2 mm, 2 kV, 0.4 mL/h, 80 mm/s, respectively. Well-aligned printed fibers with smooth surfaces were generated. In order to decrease grid void size to 200 × 200 μm^2^, the collector speed was decreased from 80 to 50 mm/s. It should be noted that precise void geometry (between aligned fiber struts) can only be achieved at reduced collector speeds. Greater speeds (e.g. >80 mm/s) result in irregular pore sizes during deposition.

Figure 4d and e show fiber diameter distributions for grids with pore sizes of 200 × 200 and 500 × 500 μm^2^, with mean fiber diameters of 12.7 ± 0.8 and 10.6 ± 1.1 μm, respectively. The variation in fiber diameter is explained due to the drawing force, jet-substrate contact time and ejected jet spreading during the EHD printing process. As the collector speed increases, the mechanical drawing force yields finer fibers. 3D rhomboid structure generation is also achievable (Fig. 4c) by changing the path of the *x-y* pre-determined program. For this, the operating parameters were kept identical to those utilised for the generation of 200 × 200 μm^2^ grids (but *x-y* for rhomboid geometry) and for this the mean fiber diameter was 10.2 ± 0.9 μm. Hence, the EHD printing technique allows on demand fabrication of 3D structures with desirable grid size, grid void (pore) size, fiber size (strut) and fiber orientation.

### Water contact angle analysis

**Figure 5:**
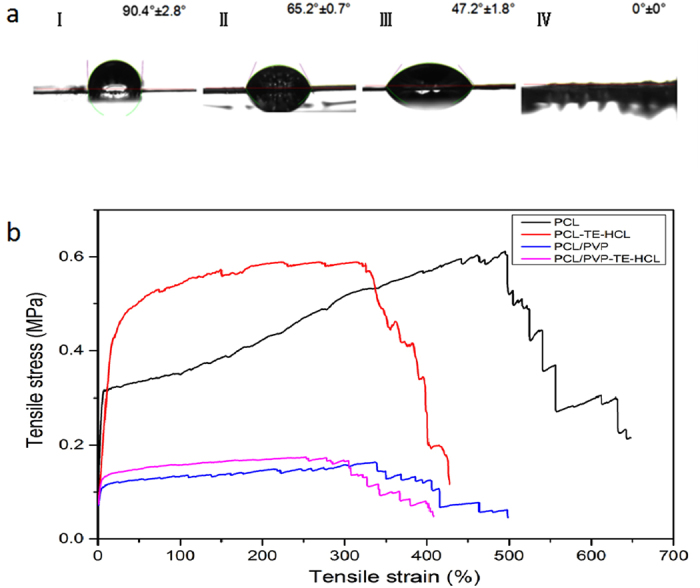
(**a**) Water contact angle on printed films comprising PCL/PVP samples with grid pore sizes of (I) 200 × 200 μm^2^ and (II) 500 × 500 μm^2^. Drug loaded patches comprising (III) PCL/PVP loaded with 1 w/w% TE-HCL with a grid pore size of 500 × 500 μm^2^ and (IV) PCL/PVP loaded 2 w/w% TE-HCL with a grid pore size of 500 × 500 μm^2^. (**b**) Stress-strain curves of four printed patch systems.

TE-HCL loaded PCL/PVP (25 w/v%) printed patches (20 layers) demonstrated interesting wetting characteristics as shown in Fig. 5a. Both TE-HCL and PVP are hydrophilic while PCL is hydrophobic. PCL/PVP printed samples possessing pore sizes of 200 × 200 μm^2^ exhibit a mean water contact angle of 90.4 ± 2.8° as shown in Fig. 5aI. The hydrophilic nature of PVP polymer coupled to the grid pore size enhances the contact area between water and printed patches. For grids possessing pore sizes of 500 × 500 μm^2^, the mean contact angle decreased from 90.4 ± 2.8 to 65.2 ± 0.7° as shown in Fig. 5aII, which indicates the grid pore size improves hydropholicity. When TE-HCL (~1 w/w% of deployed polymer system) is incorporated into PCL/PVP printed patches (grid pore size = 500 × 500 μm^2^), the contact angle decreases further from 65.2 ± 0.7 to 47.2 ± 1.8° as shown in Fig. 5aIII. Increasing the drug concentration to 2 w/w% results in complete spreading (contact angle = 0 ± 0°), as shown in Fig. 5aIV. These changes are attributed to significant hydrophilic drug coverage on the fiber surface and the large grid pore size. Combining polymeric blends (e.g. hydrophilic PVP), drug quantity and pre-designated pore size provides an opportunistic approach to enhance wetting properties of active patches and subsequently manipulate drug release behavior.

### Mechanical properties

The mechanical properties of several printed patches (Pure PCL, PCL-TE-HCL, PCL/PVP and PCL-PVP-TE-HCL) were investigated using a universal materials tester. Fig. 5b shows examples of stress-strain curves for the four printed samples. PCL and PCL-TE-HCL patches exhibit similar tensile strengths (~0.6 MPa), which suggests TE-HCL has minimal impact on this specific property. However, the tensile strain decreased from 647.5 ± 12.8 to 427.8 ± 10.3% upon the inclusion of drug. In addition, all PCL/PVP and PCL/PVP-TE-HCL printed patches possess relatively low tensile strengths (~0.18 MPa). This is attributed to differences in PVP and PCL polymeric chain network structure, similar to what has been reported previously for polymer composite systems where poor interfacial interaction and unstable phase dispersion compromise mechanical properties.

### FTIR analysis

**Figure 6:**
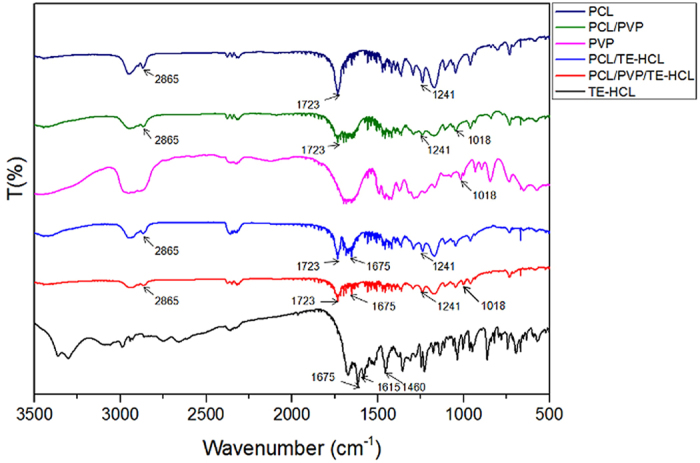
FTIR spectra of pure PCL, PCL/PVP, pure PVP, PCL/TE-HCL, PCL/PVP-TE-HCL and pureTE-HCL.

FTIR analysis was used to confirm the presence and possible interaction between patch materials. Figure 6 shows the FTIR absorbance spectra of pure PCL, PCL/PVP, pure PVP, PCL/TE-HCL, PCL/PVP/TE-HCL and pure TE-HCL. Characteristic bands for pure PCL printed fibers are observed (Fig. 6). Characteristic C=O peak at 1723 cm^−1^, CH_2_ asymmetric stretching at 2945 cm^−1^ and symmetric stretching at 2865 cm^−1^, C-O-C stretching at 1241 cm^−1^ and C-O stretching at 1170 cm^−1^ all belong to PCL^43^. In addition, CH_2_ rocking mode of PVP can be observed at 1018 cm^−1^, and a peak at ~1290 cm^−1^ refers to twisting of CH_2_ (PVP). The CH_2_ scissor mode can also be seen at 1496 cm^−1^ and the band corresponding to CH_2_ asymmetric stretching vibration occurs at 2954 cm^−1^ belonging to PVP^44^. The main bands for TE-HCL are found at 1675 cm^−1^ for C=O, 1615 cm^−1^ for C=C stretching of aromatic ring and 1460 cm^−1^ for OH bending. Characteristic peaks attributed to C-C stretching and bending, N-H bending and C-N stretching are found between 1250–1200 cm^−1^ belonging to TE-HCL^45^,^46^. Compared with the pure TE-HCL absorbance spectrum, a new peak at 1723 cm^−1^ belonging to PCL is observed for the PCL/TE-HCL patch. For the PCL/PVP composite system, peaks at 1723 cm^−1^ and 2865 cm^−1^ belong to PCL and the peak at 1018 cm^−1^ belongs to PVP. In addition, the peak at 1723 cm^−1^ belongs to PCL, the peak at 1019 cm^−1^ belongs to PVP, the peak at 1675 cm^−1^ belongs to TE-HCL in the PCL/PVP/TE-HCL printed sample absorbance spectra, which indicates successful formulation and drug encapsulation.

### Characterization of 3D EHD printed drug-loaded patches

**Figure 7:**
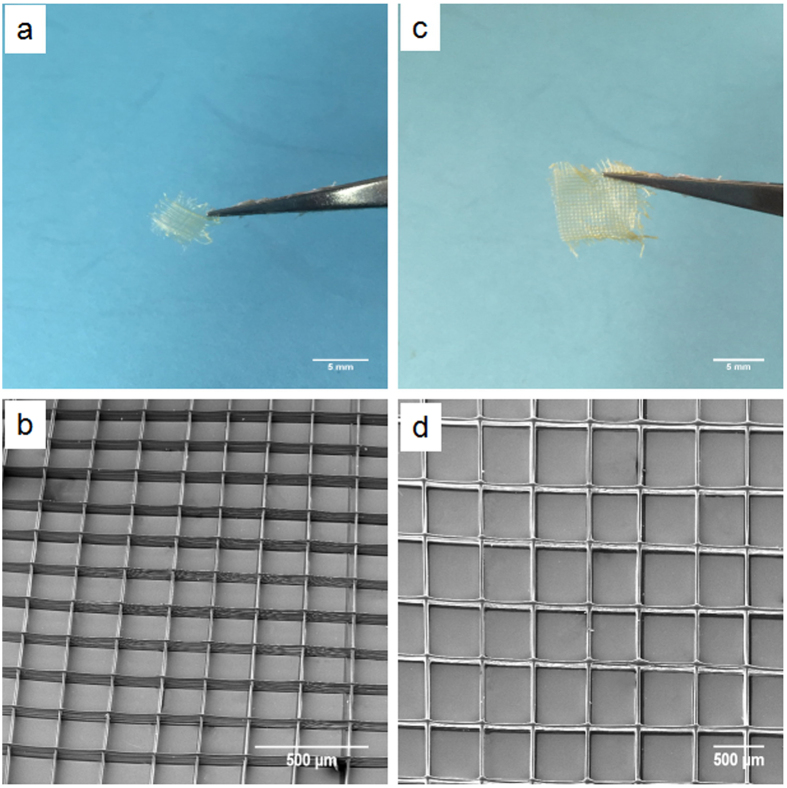
(**a**) Digital photographs of drug-loaded samples with grid pore size 200 × 200 μm^2^. (**b**) SEM images of drug-loaded samples with grid pore size 200 × 200 μm^2^. (**c**) Digital photographs of drug-loaded samples with grid pore size 500 × 500 μm^2^. (**d**) SEM images of drug-loaded samples with grid pore size 500 × 500 μm^2^.

Figure 7a and c show digital images of 3D (20 overprinted layers) aligned fiber composite (drug-loaded, 1 w/w%) patch samples. Samples possessing strut pore sizes of 200 × 200 μm^2^ (patch size = 0.5 × 0.5 cm^2^, Fig. 7a) and 500 × 500 μm^2^ (patch size = 1 × 1 cm^2^, Fig. 7c) were mechanically viable for drug release studies due to sufficient micron sized fiber overlapping to form grid type film patches^47^. The mean diameter of drug-loaded fibers in printed grids for pore size 200 × 200 μm^2^ and 500 × 500 μm^2^ were 10.8 ± 0.6 and 11.2 ± 1.4 μm, respectively. Electron micrographs of the two samples are shown in Fig. 7b (pore size 200 × 200 μm^2^) and 7d (pore size 500 × 500 μm^2^), demonstrating a clear and precise 3D hierarchical structure consisting of interconnected pores and clear and distinct layers which is not possible using conventional or recently modified ES techniques^47^.

### Patch *In-vitro* drug release

**Figure 8:**
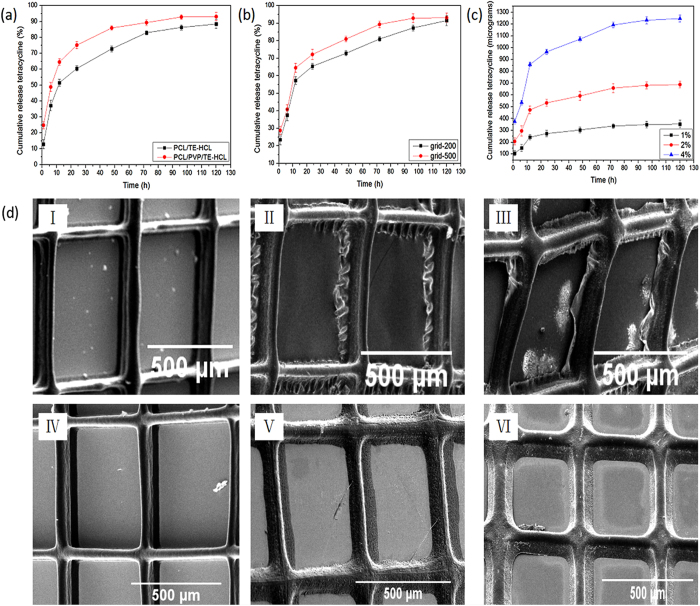
Drug releasing profiles from; (**a**) pure PCL and PCL/PVP printed patches, (**b**) grids with varying pore sizes, (**c**) various TE-HCL loading within fiber matrix and (**d**) electron micrographs of drug loaded (TE-HCL) patches comprising PCL and PCL/PVP showing changes to fiber morphology at selected time intervals over the *in vitro* release study. (I) Drug-PCL printed patch at 30 minutes (II) Drug-PCL printed patch at 60 minutes (III) Drug-PCL printed patch at 90 minutes (IV) Drug-PCL/PVP printed patch at 30 minutes (V) Drug-PCL/PVP printed patch at 60 minutes (VI) Drug-PCL/PVP printed patch at 90 minutes.

In order to investigate TE-HCL release behavior from printed patches, three comparative experiments were systematically performed in PBS (pH = 7.4) at ambient conditions. Encapsulating polymer systems were varied to demonstrate the impact of excipient material composition, which is a crucial factor in drug dosage formulation. For printing, the single step operation enables minimal excipient usage. The drug entrapment efficiency within the various filamentous matrices was relatively high as shown in Tables 1 and 2, which is in accordance with previous studies incorporating TE-HCL drug and polymeric fibers *via* ES^48^. Figure 8a shows TE-HCL release from PCL and PCL/PVP printed systems. Both samples (test patch size ~1 × 1 cm^2^) possessed pore (void) sizes of 500 × 500 μm^2^ and TE-HCL concentration was maintained at 1 w/w%. The encapsulation efficiencies for PCL/TE-HCL and PCL/PVP/TE-HCL were 95.2 ± 1.2 and 98.4 ± 0.9%, respectively. Drug release from pure PCL polymer patches was comparatively slow when compared to PCL-PVP system. 12.5 ± 2.8% of the active was released within 1 hour from PCL patches compared to 25.3 ± 1.8% of active release from the composite samples. TE-HCL release was demonstrated over a five day period with active release from pure PCL patches at a much slower rate. This is attributed to the hydrophobic nature of PCL. Blends of hydrophobic polymer (e.g. PVP) along with hydrophilic polymer enhance drug release which is evident from this study. TE-HCL release from composite fiber patches is expedited due to the rapid dissolution of PVP. Drug intercalating with PVP is also released at this stage. Active remaining within the matrix core (PCL) is released over a sustained time period (at 5 days ~88.3 ± 1.2% of the active is released).

Figure 8d shows electron micrographs of PCL-TE-HCL and PCL/PVP-TE-HCL printed patches observed at defined intervals over the *in vitro* release test period. A distinct difference in patch morphology is observed at 30, 60 and 90 minutes for both systems. Changes to both patches over the release period include spreading and broadening of polymeric fibers which become less well-defined over the release period. This is due to interaction and uptake of the release medium *via* diffusion. PCL patches are prone to hydrolytic degradation but as the polymeric is hydrophobic; fiber wrinkling is observed at printed edges (as shown in Fig. 8d I,II and III). Patches prepared with PVP polymer merge more readily (as shown in Fig. 8d IV,V and VI) which is due to rapid dissolution of the polymer in the release medium. In addition, composite films also undergo hydrolytic degradation and swelling due to diffusive mechanisms.

As shown in Fig. 8b, patch pore size has significant impact on TE-HCL (maintained at 1 w/w%) release. Pore size has minimal impact on encapsulation efficiency (~98%) for both samples as shown in Table 1. TE-HCL release (within 1 day) from printed PCL/PVP patches possessing pore sizes of 200 × 200 μm^2^ (65.4 ± 1.6%) was relatively slower compared to those with pore sizes of 500 × 500 μm^2^ (72.1 ± 2.9%). After the initial burst phase, a slow and steady release period of TE-HCL was observed up to day 5. This is due to an increased pore volume which provides a greater interfacial surface for diffusion between test medium and drug embedded fibrous matrix. The pore volume can be tailored in printed patches implying a non-excipient based strategy to control active release. When compared to 2D random fibers, 3D aligned fibers are printed into pre-designated structures with modulated pore size; both of which can be used to control the release profile of embedded drug.

The impact of TE-HCL loading (1, 2 and 4 w/w%) on its release behavior is shown in Fig. 8c and encapsulation efficiencies are shown in Table 2. The encapsulation efficiencies (97~98%) for all drug concentrations were high as minimal quantities of active were lost during the printing procedure. The patch (sample size = 1 × 1 cm^2^) pore size was maintained at 500 × 500 μm^2^. The initial burst release increased from 106.4 ± 21.2 μg to 375.2 ± 21.5 μg with increasing TE-HCL concentration from 1 to 4 w/w%. This is ascribed to greater drug molecule distribution near the fiber surface which arises due to the EHD drying process^49^. Surface assembled drug was released instantaneously upon insertion in to the test medium. After the initial burst phase, a slow and steady release rate was observed over 5 days.

Hence, the loading of TE-HCL into printed patches in a single step, must be optimized to ensure drug release is appropriate (reaching therapeutic concentration) where the release of active is sufficient to inhibit and further reduce bacterial infection over a designated time period.

In summary, EHD printing has been successfully used to print well-aligned 3D fibrous composite polymer-drug patches. The effect of various operating parameters (e.g. collector speed, flow rate, working distance and applied voltage) on fiber diameter and morphology was determined leading to process optimization. The controlled release of antimicrobial drug (TE-HCL) over a 120 hr period *in vitro* was demonstrated from a variety of printed dosage forms, all of which exhibited characteristic burst release followed by stable and sustained release. The EHD printing technique enabled size, dimension, pore volume, drug loading and patch thickness control, which is ideal for on-demand bespoke film engineering. Furthermore, minimal conventional excipient usage during printing provides an exciting opportunity to develop dosage forms for various anatomical sites and age groups where the delivery, type and quantity of conventional pharmaceutical medicines used may be challenging.

## Material and Methods

### Materials

Polycaprolactone (PCL, mean M_*w*_ = 8 × 10^4^ g/mol) and Polyvinyl pyrrolidone (PVP, mean M_w_ = 1.3 × 10^6^ g/mol) were purchased from Sigma-Aldrich Ltd., St Louis, USA. Tetracycline hydrochloride (TE-HCL) was purchased from Amresco, USA. Acetic acid and Phosphate buffer saline (PBS, PH = 7.4) were obtained from Sinopharm Chemical Reagent Co., Ltd, Shanghai, China. All materials were used as received without further purification treatment. All purchased materials were of the analytical grade.

### Solution preparation

PCL/PVP composite solutions were prepared by dissolving between 20–30 w/w% PCL/PVP (w_PCL_/w_PVP_ = 2/1) in acetic acid (at 30 °C) with continuous magnetic stirring (VELP ARE heating magnetic stirrer, Italy) for 12 h to ensure complete dissolution. Polymeric solutions were then degassed to remove any bubbles. PCL/PVP solution (w/w% = 25%, w_PCL_/w_PVP_ = 2/1) was blended with model antibiotic drug TE-HCL at different concentrations of the active (between 1 to 4 w/w%). The drug was initially dissolved in acetic acid followed by the addition of PCL and PVP. The solution was stirred at room temperature for 12 h before EHD printing. Polymer-antibiotic 3D patterned structures were generated using EHD printing.

### EHD printing 3D patterned composite patches

Figure 1a shows a schematic diagram of the custom built EHD printer and Fig. 1b shows a digital image of the actual device. The printing device consists of a stainless steel nozzle with an inner orifice diameter of ~500 μm, a high-precision programmable syringe pump (KD Scientific KDS100, USA), *X-Y-Z* motion stage and a high-voltage power supply capable of generating ~30 kV (Glassman high voltage Inc. series FC, USA). The conductive collector was mounted onto the programmable *X-Y-Z* motion stage, controlled by a mechatronics controller, allowing precise programmable movement during formulation deposition. The PCL/PVP blended formulation was loaded in a 5 ml standard syringe fitted with a needle (gauge inner diameter = 0.5 mm) and an electric potential of 1.5~3 kV was applied using a high-voltage power supply. The distance between the conductive collector and the nozzle head was varied between 2 and 8mm and the flow rate was maintained between 0.2~0.8 ml/h to ensure a stable and continuous Taylor cone for EHD printing. First, the concentration of the PCL/PVP blended solution was investigated for fabricating 3D patterned structures by EHD printing. Based on the optimized concentration, the orientation of fibers was achieved by pre-designated (programmed) movement of the *X-Y-Z* stage, and continuous fibers were deposited along the *Y*-axis to form the first layer with programmed back and forth motion. Similarly, the second layer was achieved along the *X*-axis, and the above procedure was repeated with the needle moving upward along the *Z*-axis until the patch was fabricated with multiple layers. Different pore sizes and 3D grid type patterns were achieved through uploading desired geometrical patterns directly on to the motion stage software.

In order to investigate drug release behavior from PCL/PVP-drug composite fibers, 3D grid type patches (pore size = 500 × 500 μm^2^) of pure PCL and PCL/PVP drug-loaded fibers were fabricated. Pre-determined 3D grid patterns were used (grid pore size = 200 × 200 μm^2^, 500 × 500 μm^2^) to further fabricate and assess drug release from fibrous composite patches. TE-HCL concentration within the filamentous systems was maintained at 1 w/w% as a control. For drug loading impact (on release behavior); 1, 2 and 4 w/w% antibiotic loading concentrations were selected.

### Characterization

PCL/PVP composite fibers were investigated using scanning electron microscopy (SEM) (Hitachi, Japan), optical contact angle and interface tension meter (SL200KB, Kino Industry CO. Ltd., Norcross, GA, USA) and Fourier transform infrared spectroscopy (FTIR) (IR Affinity 1, Shimadzu, Japan). SEM was used to study the size and surface morphology of the composite fibers. Fiber diameters were quantified by statistical distribution that involved a random sample of 20 fibers for each experimental condition. The mean fiber diameter (D_average_) and the standard deviation (STDEV) were determined using Equations 1 and 2.


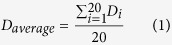



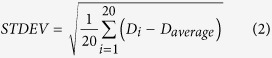


Samples for SEM analysis were deposited on microscopic glass slides and were mounted on to a specimen stub with double-sided conductive tape and then coated with Pt for 90 s at a current intensity of 25 mA (Sputter coater 108 auto, Cressington Scientific Instruments Ltd., UK) prior to analysis. SEM images were recorded at an acceleration voltage of 5 kV. Water contact angle (WCA) was measured by carefully dripping 1 μL distilled water onto patches directly deposited on to glass substrates. WCA’s were calculated by taking the mean of three separate positions for each experimental condition. Images of water-membrane interaction were obtained. Tensile tests were performed using a universal materials tester (Zwick/Roell Z020, Zwick, Germany). The selected gauge length was 10 mm, and samples were extended along the fiber axis with a 500 N load cell at a crosshead speed of 10 mm/min at the ambient temperature (24 °C). Printed PCL patches (PCL, PCL/TE-HCL, PCL/PVP and PCL/PVP/TE-HCL) possessing dimensions of 50 × 10 mm with 20 over-printed layers were assessed for mechanical properties. For each sample set, measurements were taken in triplicate. FTIR was used to examine polymeric blends and compositions within printed patches. For this, samples were prepared using the KBr pellet pressing method. 2 mg of each sample (pure PCL, composite PCL/PVP, pure PVP fibers, pure TE-HCL, PVP/TE-HCL and PCL/PVP/TE-HCL) were dispersed in 200 mg of KBr medium by grinding and then compressed into transparent pellets (pressure of 20 MPa). All pellets were then scanned with FTIR over a range of 3500–500 cm^−1^. Each spectrum was obtained using 20 scans.

### Drug release from 3D printed patches and encapsulation efficiency

Phosphate buffer saline (PBS, PH = 7.4) was used as the medium for *in vitro* drug release studies from drug loaded fibers. TE-HCL concentration in PBS was determined at 363 ± 2 nm using UV spectroscopy (Shimadzu, Japan). A linear calibration curve was established based on standard solutions with concentrations ranging from 5–100 μg/ml. All drug-release experiments were carried out with a known quantity of drug-polymer sample immersed in a sealed bottle containing 100 mL of PBS (stirred) at ambient temperature (at 25 °C). At pre-determined time intervals, the release medium was collected and centrifuged at a speed of 4200 rpm for 10 min. Then, 1 ml of supernatant was removed to determine the concentration of drug released using UV spectrophotometry and replaced with an equal volume of fresh release medium. The cumulative release of TE-HCL (α) determined using equation (3)^34^,^50^:


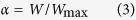


Where W is TE-HCL concentration in the solution at specified time points and W_max_ is the maximum concentration of TE-HCL in the solution. Experiments were performed in triplicate and mean values were obtained. Entrapment efficiency of all drug-polymer samples were calculated by completely dissolving all samples in acetic acid. From this the TE-HCL concentration in PBS and acetic acid was determined using UV spectroscopy. The entrapment efficiency(EE) determined using equation (4):


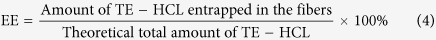


## Additional Information

**How to cite this article**: Wang, J.-C. *et al*. Preparation of active film patches *via* 3D aligned fiber electrohydrodynamic (EHD) printing. *Sci. Rep.* **7**, 43924; doi: 10.1038/srep43924 (2017).

**Publisher's note:** Springer Nature remains neutral with regard to jurisdictional claims in published maps and institutional affiliations.

## Figures and Tables

**Table 1:** Drug entrapment efficiency.

Polymer-drug system	entrapment efficiency
PCL-TE-HCL(pore size 500 × 500 μm^2^)	95.2 ± 1.2%
PCL/PVP-TE-HCL(pore size 500 × 500 μm^2^)	98.4 ± 0.9%
PCL/PVP-TE-HCL(pore size 200 × 200 μm^2^)	98.1 ± 1.6%
Drug concentration 1%w/w	

**Table 2:** Drug entrapment efficiency.

Drug TE-HCL concentration(w/w%)	entrapment efficiency
1%	97.8 ± 1.8%
2%	98.6 ± 1.0%
4%	98.5 ± 0.7%
Polymer system PCL/PVP(pore size 500 × 500 μm^2^)	

## References

[b1] Cui J., Yan Y., Wang Y. & Frank C. Drug Delivery: Templated Assembly of pH-Labile Polymer-Drug Particles for Intracellular Drug Delivery (Adv. Funct. Mater. 22/2012). Advanced Functional Materials 22, 4718–4723 (2012).

[b2] Katteboina S. & Chandrasekhar P V. S. R. Approaches for the development of solid self-emulsifying drug delivery systems and dosage forms. Asian Journal of Pharmaceutical Sciences 4, 240–253 (2009).

[b3] Kumar S., Dilbaghi N., Rani R., Bhanjana G. & Umar A. Novel Approaches for Enhancement of Drug Bioavailability. Reviews in Advanced Sciences & Engineering 2, 133–154 (2013).

[b4] Dutta R. C. & Dutta A. K. Cell-interactive 3D-scaffold; advances and applications. Biotechnology Advances 27, 334–339 (2009). 19232387 10.1016/j.biotechadv.2009.02.002

[b5] Cornelsen M. . Infiltration of 3D printed tricalciumphosphate scaffolds with biodegradable polymers and biomolecules for local drug delivery. Biomedical Engineering 58, 3234–3234 (2013). 10.1515/bmt-2013-4090 24042685

[b6] Hu X. . Electrospinning of polymeric nanofibers for drug delivery applications. Journal of Controlled Release 185, 12–21 (2014). 24768792 10.1016/j.jconrel.2014.04.018

[b7] Fang F. . In *IOP Conference Series Materials* *Science and Engineering* (2011).

[b8] Rao K. V. R. & Devi K. P. Swelling controlled-release systems: recent developments and applications. International Journal of Pharmaceutics 48, 1–13 (1988).

[b9] Correia D. M. . Influence of electrospinning parameters on poly(hydroxybutyrate) electrospun membranes fiber size and distribution. Polymer Engineering & Science 54, 1608–1617 (2014).

[b10] Hekmati A. H., Rashidi A., Ghazisaeidi R. & Drean J. Y. Effect of needle length, electrospinning distance, and solution concentration on morphological properties of polyamide-6 electrospun nanowebs. Textile Research Journal 83, 1452–1466 (2013).

[b11] Wang C., Zhang W., Huang Z. H., Yan E. Y. & Su Y. H. Effect of concentration, voltage, take‐over distance and diameter of pinhead on precursory poly (phenylene vinylene) electrospinning. Pigment & Resin Technology 35, 278–283 (2006).

[b12] Liu H. Y., Xu L., Tang X. P. & Si N. Fabrication of Aligned PAN Nanofiber by Electrospinning with Parallel Electrode. Advanced Materials Research 905, 19–22 (2014).

[b13] Yong-Ze . Fabrication of hierarchical polycaprolactone/gel scaffolds via combined 3D bioprinting and electrospinning for tissue engineering. Advances in Manufacturing 2, 231–238 (2014).

[b14] Lee J. W. & Cho D. W. 3D Printing technology over a drug delivery for tissue engineering. Current Pharmaceutical Design 21, 1606–1617 (2015). 25594413 10.2174/1381612821666150115125324

[b15] Trombetta R., Inzana J. A., Schwarz E. M., Kates S. L. & Awad H. A. 3D Printing of Calcium Phosphate Ceramics for Bone Tissue Engineering and Drug Delivery. Annals of Biomedical Engineering, 1–22 (2016). 27324800 10.1007/s10439-016-1678-3 PMC5173433

[b16] Xing J. F., Zheng M. L. & Duan X. M. Two-photon polymerization microfabrication of hydrogels: an advanced 3D printing technology for tissue engineering and drug delivery. Chemical Society Reviews 44, 5031–5039 (2015). 25992492 10.1039/c5cs00278h

[b17] Prasad L. K. & Smyth H. 3D Printing technologies for drug delivery: a review. Drug Development and Industrial Pharmacy 42, 1–35 (2016). 26625986 10.3109/03639045.2015.1120743

[b18] Hirschbiel A. F. . Photolithographic Patterning of 3D-Formed Polycarbonate Films for Targeted Cell Guiding. Advanced Materials 27, 2621–2626 (2015). 25787094 10.1002/adma.201500426

[b19] Hao J. . Three-dimensional graphene layers prepared by a gas-foaming method for supercapacitor applications. Carbon 94, 879–887 (2015).

[b20] Shin K. H., Jo I. H., Kim S. E., Koh Y. H. & Kim H. E. Nonsolvent induced phase separation (NIPS)-based 3D plotting for 3-dimensionally macrochanneled poly(ε-caprolactone) scaffolds with highly porous frameworks. Materials Letters 122, 348–351 (2014).

[b21] Sa M. W. & Kim J. Y. Design of multi-scaffold fabrication system for various 3D scaffolds. Journal of Mechanical Science & Technology 27, 2961–2966 (2013).

[b22] Hamid Q. . Fabrication of 3D scaffolds using precision extrusion deposition with an assisted cooling device. Biofabrication 3, 335–344 (2011). 10.1088/1758-5082/3/3/034109 21727312

[b23] Huang Y. A. . Electrohydrodynamic direct-writing. Nanoscale 5, 12007–12017 (2013). 24057297 10.1039/c3nr04329k

[b24] Li J. L. . Fabrication of three-dimensional porous scaffolds with controlled filament orientation and large pore size via an improved E-jetting technique. Journal of Biomedical Materials Research Part B Applied Biomaterials 102, 651–658 (2014). 10.1002/jbm.b.33043 24155124

[b25] Karuppuswamy P., Venugopal J. R., Navaneethan B., Laiva A. L. & Ramakrishna S. Polycaprolactone nanofibers for the controlled release of tetracycline hydrochloride. Materials Letters 141, 180–186 (2015).

[b26] Salerno C., Carlucci A. M. & Bregni C. Study of *in vitro* drug release and percutaneous absorption of fluconazole from topical dosage forms. Aaps Pharmscitech 11, 986–993 (2010). 20521179 10.1208/s12249-010-9457-1 PMC2902339

[b27] Kenawy E., Abdel-Hay F. I., El-Newehy M. H. & Wnek G. E. Processing of Polymer Nanofibers Through Electrospinning as Drug Delivery Systems. Textile Research Journal 78, 45–52 (2008).

[b28] Samprasit W. . Fast releasing oral electrospun PVP/CD nanofiber mats of taste-masked meloxicam. International Journal of Pharmaceutics 487, 213–222 (2015). 25899284 10.1016/j.ijpharm.2015.04.044

[b29] Karataş A., Algan A. H., Pekel-Bayramgil N., Turhan F. & Altanlar N. Ofloxacin Loaded Electrospun Fibers for Ocular Drug Delivery: Effect of Formulation Variables on Fiber Morphology and Drug Release. Current Drug Delivery 13, 11 (2015). 10.2174/1567201812666151030162258 26521656

[b30] Kadam R. S. H. & Design V. J. of Buccal Drug Delivery System for a Poorly Soluble Drug. Asian Journal of Pharmaceutical & Clinical Research 2, 49–53 (2009).

[b31] Rai B., Teoh S. H., Hutmacher D. W., Cao T. & Ho K. H. Novel PCL-based honeycomb scaffolds as drug delivery systems for rhBMP-2. Biomaterials 26, 3739–3748 (2005). 15621264 10.1016/j.biomaterials.2004.09.052

[b32] Williams J. M. . Bone tissue engineering using polycaprolactone scaffolds fabricated via selective laser sintering. Biomaterials 26, 4817–4827 (2005). 15763261 10.1016/j.biomaterials.2004.11.057

[b33] Haeyong K. . A novel degradable polycaprolactone networks for tissue engineering. Biomaterials 24, 801–808 (2003). 12485798 10.1016/s0142-9612(02)00370-8

[b34] Croisier F., Atanasova G., Poumay Y. & Jérôme C. Polysaccharide-Coated PCL Nanofibers for Wound Dressing Applications. Advanced Healthcare Materials 3, 2032–2039 (2014). 25263074 10.1002/adhm.201400380

[b35] Rasekh M. . Electrospun PVP–indomethacin constituents for transdermal dressings and drug delivery devices. International Journal of Pharmaceutics 473, 95–104 (2014). 24997411 10.1016/j.ijpharm.2014.06.059

[b36] Ranjha N. M., Khan I. U. & Naseem S. Encapsulation and characterization of flurbiprofen loaded poly(є-caprolactone)–poly(vinylpyrrolidone) blend micropheres by solvent evaporation method. Journal of Sol-Gel Science and Technology 50, 281–289 (2009).

[b37] Zahedi P. & Fallah-Darrehchi M. Electrospun egg albumin-PVA nanofibers containing tetracycline hydrochloride: Morphological, drug release, antibacterial, thermal and mechanical properties. Fibers & Polymers 16, 2184–2192 (2015).

[b38] Obaidat R. M., Bader A., Alrajab W., Sheikha G. A. & Obaidat A. A. Preparation of Mucoadhesive Oral Patches Containing Tetracycline Hydrochloride and Carvacrol for Treatment of Local Mouth Bacterial Infections and Candidiasis. Scientia Pharmaceutica 79, 197–212 (2011). 21617783 10.3797/scipharm.1004-18 PMC3097507

[b39] El-R K. . Release of tetracycline hydrochloride from electrospun poly(ethylene-co-vinylacetate), poly(lactic acid), and a blend. Journal of Controlled Release 81, 57–64 (2002). 11992678 10.1016/s0168-3659(02)00041-x

[b40] Sencadas V., Ribeiro C., Nunes-Pereira J., Correia V. & Lanceros-Méndez S. Fiber average size and distribution dependence on the electrospinning parameters of poly(vinylidene fluoride–trifluoroethylene) membranes for biomedical applications. Applied Physics A 109, 685–691 (2012).

[b41] Volova T. . Electrospinning of polyhydroxyalkanoate fibrous scaffolds: effects on electrospinning parameters on structure and properties. Journal of Biomaterials Science Polymer Edition 25, 370–393 (2013). 24295429 10.1080/09205063.2013.862400

[b42] Hellmann C., Belardi J. & Dersch R. High Precision Deposition Electrospinning of nanofibers and nanofiber nonwovens. Polymer 50, 1197–1205 (2009).

[b43] Huang Y. P., Chen G. M., Luo X. L. & De-Zhu M. A. On the Non-isothermal Crystallization of PCL by FTIR. *Chinese Journal of Applied Chemistry* (2003).

[b44] Abdelghany A. M., Mekhail, Abdelrazek E. M. & Aboud M. M. Combined DFT/FTIR structural studies of monodispersed PVP/Gold and silver nano particles. Journal of Alloys & Compounds 646, 326–332 (2015).

[b45] Caroni A. L. P. F., Lima C. R. M. D., Pereira M. R. & Fonseca J. L. C. Tetracycline adsorption on chitosan: A mechanistic description based on mass uptake and zeta potential measurements. Colloids & Surfaces B Biointerfaces 100, 222–228 (2012). 22771527 10.1016/j.colsurfb.2012.05.024

[b46] Jin K., Liu H., Zheng Y. M., Qu J. & Chen J. P. Systematic study of synergistic and antagonistic effects on adsorption of tetracycline and copper onto a chitosan. Journal of Colloid & Interface Science 344, 117–125 (2010). 20092824 10.1016/j.jcis.2009.11.049

[b47] Liu Y. Y. . Dual drug spatiotemporal release from functional gradient scaffolds prepared using 3D bioprinting and electrospinning. Polymer Engineering & Science, 170–177 (2015).

[b48] Alhusein N., Blagbrough I. S. & De Bank P. A. Electrospun matrices for localised controlled drug delivery: release of tetracycline hydrochloride from layers of polycaprolactone and poly(ethylene-co-vinyl acetate). Drug Deliv Transl Res 2, 477–488, doi: 10.1007/s13346-012-0106-y (2012). 25787326

[b49] Wang X. . Drug distribution within poly(ɛ-caprolactone) microspheres and *in vitro* release. Journal of Materials Processing Technology 209, 348–354 (2009).

[b50] Adnadjevic B., Jovanovic J. & Drakulic B. Isothermal kinetics of (E)-4-(4-metoxyphenyl)-4-oxo-2-butenoic acid release from poly(acrylic acid) hydrogel. Thermochimica Acta 466, 38–48 (2007).

